# Animal Exposure and Human Plague, United States, 1970–2017

**DOI:** 10.3201/eid2512.191081

**Published:** 2019-12

**Authors:** Stefanie B. Campbell, Christina A. Nelson, Alison F. Hinckley, Kiersten J. Kugeler

**Affiliations:** Centers for Disease Control and Prevention, Fort Collins, Colorado, USA

**Keywords:** animals, animal exposure, One Health, human plague, Yersinia pestis, bacteria, fleas, rodents, vector-borne infections, zoonoses, United States

## Abstract

Since 1970, >50% of patients with plague in the United States had interactions with animals that might have led to infection. Among patients with pneumonic plague, nearly all had animal exposure. Improved understanding of the varied ways in which animal contact might increase risk for infection could enhance prevention messages.

Plague is a rare, life-threatening zoonosis caused by *Yersinia pestis* that occurs globally in discrete foci, including the western United States (*1*). The bacterium is maintained in an enzootic cycle of rodents and their fleas (*2*). Periodically, the cycle intensifies, leading to epizootic events characterized by localized small mammal die-offs. During epizootics, the risk for incidental human infection increases (*2*). Humans are exposed to *Y. pestis* most commonly through flea bites but also through contact with tissues of infected animals or inhalation of infectious droplets.

Clinical manifestations of plague in humans are associated with route of exposure. Primary pneumonic plague, the most severe and rapidly fatal form of the disease, occurs after direct inhalation of infectious droplets coughed by infected animals or humans (*3*). Human exposure to *Y. pestis* results from direct and indirect interactions with animals. Improved understanding of the role of animals in human exposure to *Y. pestis* may foster more refined prevention messages in plague-endemic areas.

## The Study

Plague is a nationally notifiable condition in the United States (*4*). State and local health jurisdictions report human cases to the Centers for Disease Control and Prevention. Case records typically include supplemental information on possible sources of exposure, clinical course, and outcome. We reviewed data from all reported human plague cases during 1970–2017 that were characterized by a clinically compatible illness and presumptive or confirmatory laboratory evidence as defined previously (*5*). For this analysis, we considered only the primary clinical manifestation of illness.

We created a data extraction tool to capture details on patient–animal interactions in the 2 weeks preceding illness onset. If animal exposure had occurred, we classified the type of animal(s) involved as domestic or wild and the interactions as directly or indirectly associated with exposure to *Y. pestis*. We grouped animal exposures into categories based on authors’ judgment regarding risk for transmission. From high-risk to low-risk, the categories were animal bite, scratch, lick, or cough; skinning of a deceased animal; providing care to or handling a sick or deceased animal; co-sleeping; casual handling or touching; and other (walking, feeding, or contact type unspecified). If a patient had >1 animal interaction, the interaction recorded is that of the higher-risk category.

During 1970–2017, a total of 482 human plague cases were reported in the United States. Median case-patient age was 31 (range <1–94) years; 58% were male patients (Table 1). Bubonic plague was the predominant primary clinical manifestation of illness (n = 364, 76%), followed by septicemic plague (n = 91, 19%) and pneumonic plague (n = 15, 3%) (Table 1). Outcomes were known for 465 patients; 65 (14%) reportedly died from their illness.

**Table 1:** Characteristics of reported human plague case-patients, United States, 1970–2017*

Characteristic	Total	Animal exposure before illness	
		Yes	No
Case-patients	482 (100)	258 (54)	224 (46)
Sex			
M	278 (58)	152 (59)	126 (56)
F	204 (42)	106 (41)	98 (44)
Median age, y (range)	31 (<1–94)	33 (2–85)	24 (<1–94)
Race/ethnicity†‡			
White	220 (46)	135 (52)	85 (38)
American Indian/Alaska Native	114 (24)	52 (20)	62 (28)
Not specified	94 (20)	45 (17)	49 (22)
Hispanic	51 (11)	25 (10)	26 (12)
Asian	3 (<1)	1 (<1)	2 (1)
Primary clinical form†			
Bubonic	364 (76)	200 (78)	164 (73)
Septicemic	91 (19)	41 (16)	50 (22)
Pneumonic	15 (3)	13 (5)	2 (<1)
Pharyngeal	3 (<1)	2 (<1)	1 (<1)
Gastrointestinal	2 (<1)	1 (<1)	1 (<1)
Other and unknown	7 (1)	1 (<1)	6 (3)
Died	65 (14)	40 (16)	25 (11)
Known flea bite	104 (22)	49 (19)	55 (25)
State of exposure			
New Mexico	253 (52)	124 (48)	129 (58)
Colorado	66 (14)	44 (17)	22 (10)
Arizona	62 (13)	33 (13)	29 (13)
Other and unknown	101 (21)	57 (22)	44 (20)

*Values are no. (%) unless otherwise indicated. †Indicates statistically significant difference (α = 0.05) in proportion with characteristic between patients with animal exposure and those without animal exposure. ‡Several established race and ethnicity categories were absent among reported case-patient records and therefore not included.

Animal exposure that was plausibly related to plague transmission was identified in 258 (54%) records. The median case-patient age was greater among those with animal exposure (33 years) than those without animal exposure (24 years) (p<0.05). The frequency of known flea bite and mortality rate did not differ between patients with animal exposures and those without animal exposures (Table 1). After peaking in the 1980s, frequency of human plague decreased (Figure). However, the proportion of plague cases with animal exposure seemingly increased over time, from 52% in the years before 2000 to 63% since 2000 (p = 0.07) (Figure).

**Figure:**
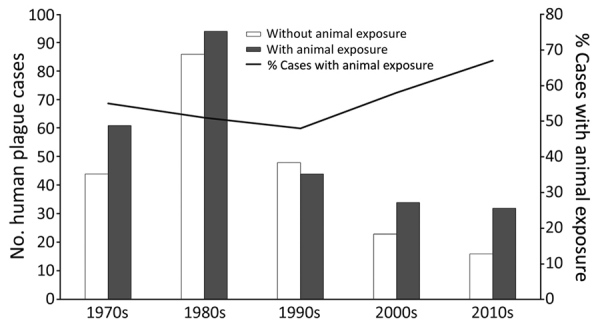
Frequency of animal exposure among human plague cases, by decade, United States, 1970–2017.

Of the 258 plague patients with animal exposures, 154 (60%) had contact with domestic animals before illness, including 121 with dogs and 102 with cats. The types of interactions included casual handling or touching (n = 55, 36%); co-sleeping (n = 31, 20%); caring for or handling a sick or dead animal (n = 29, 19%); bite, scratch, lick, or cough (n = 20, 13%); or other (n = 19, 12%) (Table 2). Among those with domestic animal contact, 65 (42%) had exposure to a domestic animal that brought home dead wild animals and 21 (14%) to a domestic animal with evidence of fleas.

**Table 2:** Animal type and nature of interaction for 258 human plague case-patients with identified animal exposures, United States, 1970–2017

Category of animal interaction	Domestic animal, n = 154, no. (%)*	Wild animal, n = 134, no. (%)*
Bite, scratch, lick, cough	20 (13)	2 (1)
Skinning	0 (0)	54 (40)
Handling a sick or dead animal	29 (19)	37 (24)
Co-sleeping	31 (20)	0 (0)
Casual handling or touching	55 (36)	29 (22)
Other†	19 (12)	12 (9)

*Interaction ordered from highest to lowest risk. †Walking, feeding, or nature of interaction not specified.

A total of 134 (52%) patients had exposure to wild animals before illness. Common wild animal exposures were to sciurid rodents (e.g., squirrels, prairie dogs, gophers) (n = 58), lagomorphs (n = 50), other rodents (n = 40), wild carnivores (n = 15), and cervids (e.g., antelope, deer) (n = 9). Types of interactions identified were skinning (n = 54, 40%); handling a sick or dead animal (n = 37, 24%); casual handling or touching (n = 29, 22%); other type of contact (n = 12, 9%); and bite, scratch, lick, or cough (n = 2, 1%) (Table 2). Wild animal interactions were generally higher-risk, more direct exposures.

Pneumonic plague occurred more frequently among patients with animal exposure (n = 13, 5%) than among those without animal exposure (n = 2, 1%) (p<0.05); most patients had a history of contact with domestic animals (n = 11, 73%). Of 6 pneumonic plague cases associated with occupational exposures, 5 were among veterinarians or veterinary technicians providing care to plague-infected animals. The proportions of bubonic (n = 205, 77% vs. n = 162, 75%) and septicemic (n = 42, 16% vs. n = 49, 23%) cases were similar between patients with and without these exposures (Table 1).

## Conclusions

More than 50% of patients in the United States with plague since 1970 had animal interaction that might have directly or indirectly led to their exposure to *Y. pestis*. Animals are associated with human plague transmission in varied ways, ranging from direct exposure, such as caring for a plague-infected animal, to more subtle indirect encounters with infected fleas, such as by co-sleeping with a flea-infested pet in an area with epizootic plague. Nearly all patients with pneumonic plague had animal interaction before illness, and several occurred in an occupational setting. Although the frequency of human plague in the United States has decreased, the proportion of human cases potentially related to animal exposure has concomitantly increased.

Cat-associated and wild animal–associated human plague have been documented in previous reports (*6*–*9*). More recently, the severity of plague illness in dogs and the role these animals might play in human plague have been recognized (*10*). A cluster of pneumonic plague in Colorado was linked to a dog with pneumonic plague (*11*), and a recent case of canine plague resulted in the potential exposure of >116 persons at a veterinary clinic (*12*). Gould et al. found that co-sleeping with a dog occurred more frequently among human plague case-patients than among neighborhood controls (*13*).

Limitations of our analysis include the possibility that human plague cases might have gone undiagnosed and thus were not captured. Our findings might underrepresent animal-associated plague because case records contain variable levels of detail. Thus, some patients might have had animal exposures that were not captured. In many instances, we could not determine which exposure contributed to human illness, if any at all. Therefore, this analysis is meant to describe the potential rather than definitive scope of animal-related human plague.

This report offers perspective on frequency and diversity of animal interaction as possible means of human exposure to *Y. pestis* in the United States. Given that most human plague worldwide is caused by flea bites, animal-associated prevention messages have been geared toward hunters and trappers, including the use of gloves when handling or skinning wild animals. Our findings highlight One Health–oriented opportunities to maximize plague prevention through communication with veterinarians in plague-endemic areas. Veterinarians play an integral role in plague prevention for animals and humans by increasing use of flea prevention products, promoting basic precautions among pet owners caring for sick pets, and encouraging use of appropriate personal protective equipment in the veterinary community.

## References

[1] Pollitzer R. Plague. World Health Organization monograph series; 1954 [cited 2019 Jan 15]. http://apps.who.int/iris/bitstream/10665/41628/1/WHO_MONO_22.pdf?ua=1

[2] Gage KL, Kosoy MY. Natural history of plague: perspectives from more than a century of research. Annu Rev Entomol. 2005;50:505–28. 10.1146/annurev.ento.50.071803.130337 15471529

[3] Mandell GL, Bennett JE, Dolin R, editors. Principles and practice of infectious diseases. Philadelphia: Churchill Livingstone Elsevier; 2010.

[4] Centers for Disease Control and Prevention. National notifiable diseases surveillance system. Plague (*Yersinia pestis*) 1996 case definition [cited 2019 Sep 4]. https://wwwn.cdc.gov/nndss/conditions/plague/case-definition/1996

[5] Kugeler KJ, Staples JE, Hinckley AF, Gage KL, Mead PS. Epidemiology of human plague in the United States, 1900-2012. Emerg Infect Dis. 2015;21:16–22. 10.3201/eid2101.140564 25529546 PMC4285253

[6] Gage KL, Dennis DT, Orloski KA, Ettestad P, Brown TL, Reynolds PJ, et al. Cases of cat-associated human plague in the Western US, 1977-1998. Clin Infect Dis. 2000;30:893–900. 10.1086/313804 10852811

[7] Eidson M, Tierney LA, Rollag OJ, Becker T, Brown T, Hull HF. Feline plague in New Mexico: risk factors and transmission to humans. Am J Public Health. 1988;78:1333–5. 10.2105/AJPH.78.10.1333 3421391 PMC1349433

[8] Wong D, Wild MA, Walburger MA, Higgins CL, Callahan M, Czarnecki LA, et al. Primary pneumonic plague contracted from a mountain lion carcass. Clin Infect Dis. 2009;49:e33–8. 10.1086/600818 19555287

[9] Doll JM, Zeitz PS, Ettestad P, Bucholtz AL, Davis T, Gage K. Cat-transmitted fatal pneumonic plague in a person who traveled from Colorado to Arizona. Am J Trop Med Hyg. 1994;51:109–14. 10.4269/ajtmh.1994.51.109 8059908

[10] Nichols MC, Ettestad PJ, Vinhatton ES, Melman SD, Onischuk L, Pierce EA, et al. *Yersinia pestis* infection in dogs: 62 cases (2003-2011). J Am Vet Med Assoc. 2014;244:1176–80. 10.2460/javma.244.10.1176 24786165

[11] Runfola JK, House J, Miller L, Colton L, Hite D, Hawley A, et al.; Centers for Disease Control and Prevention (CDC). Outbreak of human pneumonic plague with dog-to-human and possible human-to-human transmission—Colorado, June–July 2014. MMWR Morb Mortal Wkly Rep. 2015;64:429–34. 25928467 PMC4584809

[12] Schaffer PA, Brault SA, Hershkowitz C, Harris L, Dowers K, House J, et al. Pneumonic plague in a dog and widespread potential human exposure in a veterinary hospital, United States. Emerg Infect Dis. 2019;25:800–3. 10.3201/eid2504.181195 30882315 PMC6433021

[13] Gould LH, Pape J, Ettestad P, Griffith KS, Mead PS. Dog-associated risk factors for human plague. Zoonoses Public Health. 2008;55:448–54. 18489541 10.1111/j.1863-2378.2008.01132.x

